# Effect of different plant communities on NO_2_ in an urban road greenbelt in Nanjing, China

**DOI:** 10.1038/s41598-023-30488-0

**Published:** 2023-02-28

**Authors:** Anqi Dai, Congzhe Liu, Yaou Ji, Qianqian Sheng, Zunling Zhu

**Affiliations:** 1 College of Landscape Architecture, Nanjing Forestry University, Nanjing, 210037 China; 2 Co-Innovation Center for Sustainable Forestry in Southern China, Nanjing Forestry University, Nanjing, 210037 China; 3 College of Art and Design, Nanjing Forestry University, Nanjing, 210037 China

**Keywords:** Urban ecology, Environmental impact

## Abstract

As an important part of urban ecosystems, plants can reduce NO_2_ concentrations in the air. However, there is little evidence of the effects of different plant communities on NO_2_ concentrations in street-scale green spaces. We used a multifunctional lifting environmental detector to investigate the impact of environmental factors and small plant communities on NO_2_ concentrations in street green spaces during the summer and winter in Nanjing, China. The results showed that temperature, atmospheric pressure, and noise were significantly (*P* < 0.05) correlated with seasonal changes, temperature and humidity significantly (*P* < 0.01) influenced NO_2_ concentrations in winter and summer, and the average NO_2_ concentration in summer was generally higher than in winter. By comparing NO_2_ concentrations in different plant community structures and their internal spaces, we found that the plant community structure with tree-shrub-grass was more effective in reducing pollution. These findings will help predict the impact of plant communities on NO_2_ concentrations in urban streets and help city managers and planners effectively reduce NO_2_ pollution.

## Introduction

Air pollution is a major global health problem. As the world’s largest developing country, China’s economic growth has been accompanied by a rapid and large increase in NO_2_ concentration^1^. In the past, China was committed to reducing atmospheric NO_2_ emissions at the national level^2^. For example, the NO_2_ emission reduction strategy formulated in the 13th Five-Year Plan (2016–2020) has markedly contributed to reducing NO_2_ emissions^3^. However, the current study shows that spatially clustered population exposure to NO_2_ still exists in some regions and provincial capitals of large urban agglomerations in China^4^. As an important tropospheric trace gas and precursor of photochemical smog, NO_2_ accumulation causes serious air pollution^5^ and has a significant ecological impact on the surrounding environment^6^. In addition, long-term exposure to NO_2_ is deleterious to human health^7^ or even causes death^8^. For example, Hu et al.^9^ showed that NO_2_ increases the mortality of individuals with cardiovascular and respiratory diseases.

The present study paid more attention to the tropospheric NO_2_ column than the surface. However, ground NO_2_ concentration is more closely associated with anthropogenic emissions, is considerably related to transportation^10^, and directly affects human health^2^.

Although the key to reducing NO_2_ concentrations in the air is to reduce emissions^11^, it is necessary to explore alternative solutions because of the difficulty of solving air pollution by completely controlling pollution sources. As an important part of urban ecosystems, plants have received widespread attention because of their ability to evaluate air pollution status and as an effective indicator of air pollution^12^ and reduce NO_2_ concentrations through multiple pathways^13^. Researchers tend to conduct such research in three ways. One is to select locally common road-grown plants and screen plant species that effectively alleviate NO_2_ concentrations through laboratory fumigation^13,14^. Second, ENVI-Met, FLUENT, MISKAM, OSPM, and other air pollutant diffusion models have been used to conduct numerical simulations^15,16^. The third is to explore the purification effect of vegetation by measuring the NO_2_ concentration in plant communities in the field^17,18^. Using these methods, we believe that measuring the change in NO_2_ concentration in the real environment is more conducive to exploring the purification effect of plants under various complex conditions. Interestingly, field studies have shown that the purifying effect of plants on NO_2_ concentrations depends on, and often contradicts, the climatic conditions at the time of research and the vegetation type and structure^19–21^. These studies mainly focused on urban green spaces, such as forests and parks^22,23^, and were less concerned with whether street-scale green spaces can reduce NO_2_ concentrations. However, this green space is in direct contact with NO_2_ from traffic emissions, and we feel its importance may be overlooked.

Based on the subject matter discussed above, our current study focused on common street plant species, such as street trees, shrubs, and herbs. By studying different plant communities in summer (vigorous plant growth) and winter (poor plant growth) in Nanjing, we analyzed the influence of different plant communities on the NO_2_ concentration and its variation in the horizontal and vertical directions under careful consideration of street meteorological factors.

## Materials and methods

### Overview of the study area

**Figure 1:**
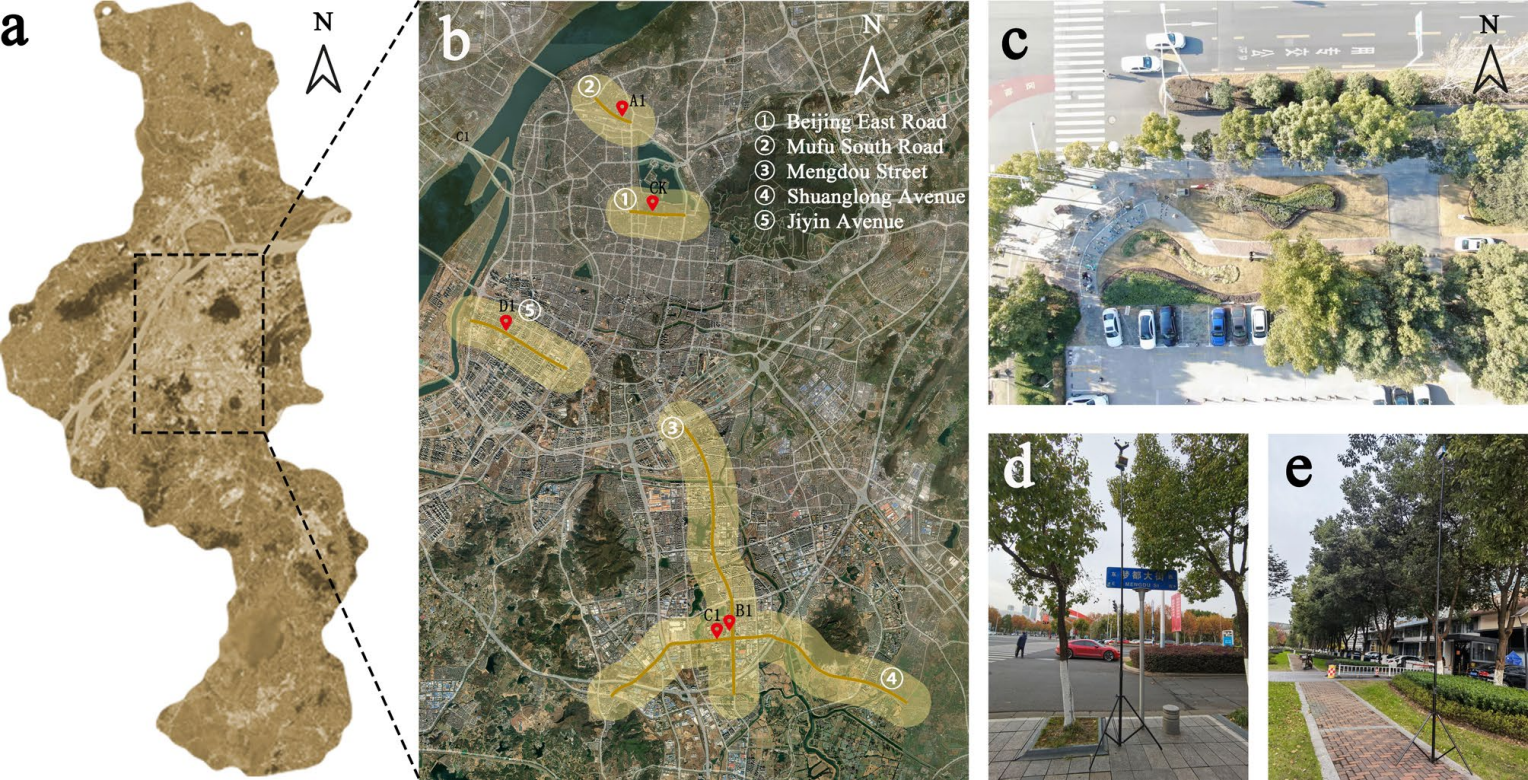
(**a**) Nanjing, Jiangsu Province, China, (**b**) location of five roads and five sampling sites in Nanjing, (**c**) aerial photo of sampling point of Mengdu Street, (**d**) sampler set at 6 m height of sidewalk in Mengdu Street, (**e**) sampler set at 6 m height of green space in Mengdu Street. (The base images for (**a**) and (**b**) are from Google Maps. Version number: 11.60.0703. URL link: https://www.google.com.hk/maps/place/%E4%B8%AD%E5%9B%BD%E6%B1%9F%E8%8B%8F%E7%9C%81%E5%8D%97%E4%BA%AC%E5%B8%82/@32.0554234,118.67779,59275m/data=!3m1!1e3!4m5!3m4!1s0x35b58c9b668dcd83:0x8ffbb60b79df1b06!8m2!3d32.0583799!4d118.79647!5m1!1e4?hl=zh-CN).

Nanjing is the capital of Jiangsu Province. It is located in the southwest of Jiangsu Province and the lower reaches of the Yangtze River, with latitude 31°14–32°37 N and longitude 118°22–119°14′ E. In recent years, road construction in Nanjing has developed rapidly. There are 445 roads in the urban area, including 75 main roads, 77 secondary roads, and 303 branch roads. The total length of roads in the city has reached 734, and 670.10 km are green, with a greening rate of 91.29%. By 2016, the road density had reached 5.39 km/km^2^, and the road area ratio (road area/built-up area) had reached 19.8%. Based on the investigation of all the roads in the urban area of Nanjing, we selected five roads in four main urban areas of Nanjing (Jianye District, Xuanwu District, Gulou District, and Jiangning District) as the research sites (Fig. 1). On the basis, we conducted a 5% random sampling survey on the plants of these 5 roads and selected widely represented plant communities (with trees, shrubs, and grasslands as the main vegetation types of the study).

### Sample site selection and plant community characteristics

We selected five sampling sites in five plant communities along five roads in Nanjing. Each site was represented by its unique plant species. As shown in Table 1, according to the different plant community types, the study divided the plant configuration patterns into five types: a control point (hard pavement square), lawn, arbor-grass, arbor-shrub, and arbor-shrub-grass. The control point CK was located in Beijing East Road, lawn A1 in Mufu South Road, arbor-grass community B1 in Shuanglong Avenue, arbor-shrub community C1 in Jiyin Avenue, and arbor-shrub community D1 in Mengdu Street.

**Table 1:** Characteristics of the plant community structure in the street green spaces.

Structure of plant community	Street	Street tree green belts species
CK: control point	Beijing East Road	–

A1: lawn	Mufu South Road	*Cynodon dactylon*

B1: arbor-grass	Shuanglong Avenue	*Albizia julibrissin* + *Ginkgo biloba* + *Cinnamomum camphora* + *Ligustrum lucidum* + *Swida wilsoniana* + *Prunus cerasifera *− *C. dactylon*

C1: arbor-shrub	Jiyin Avenue	*Koelreuteria paniculata* + *Osmanthus fragran* + *Lagerstroemia indica* + *Prunus serrulata* + *L. lucidum* + *Photinia serratifolia *− *Euonymus japonicus '*Aurea-marginatus*'* + *Viburnum odoratissimum* + *Ligustrum* × *vicaryi* + *Nerium oleander *− *C. dactylon*

D1: arbor-shrub-grass	Mengdu Street	*C. camphora* + *O. fragrans* + *Ginkgo biloba* + *Acer palmatum *− *Loropetalum chinense* var. *rubrum* + *Rhododendron simsii* + *Pittosporum tobira* + *Photinia* × *fraseri *− *Ophiopogon bodinieri* + *C. dactylon*

### Data collection

**Figure 2:**
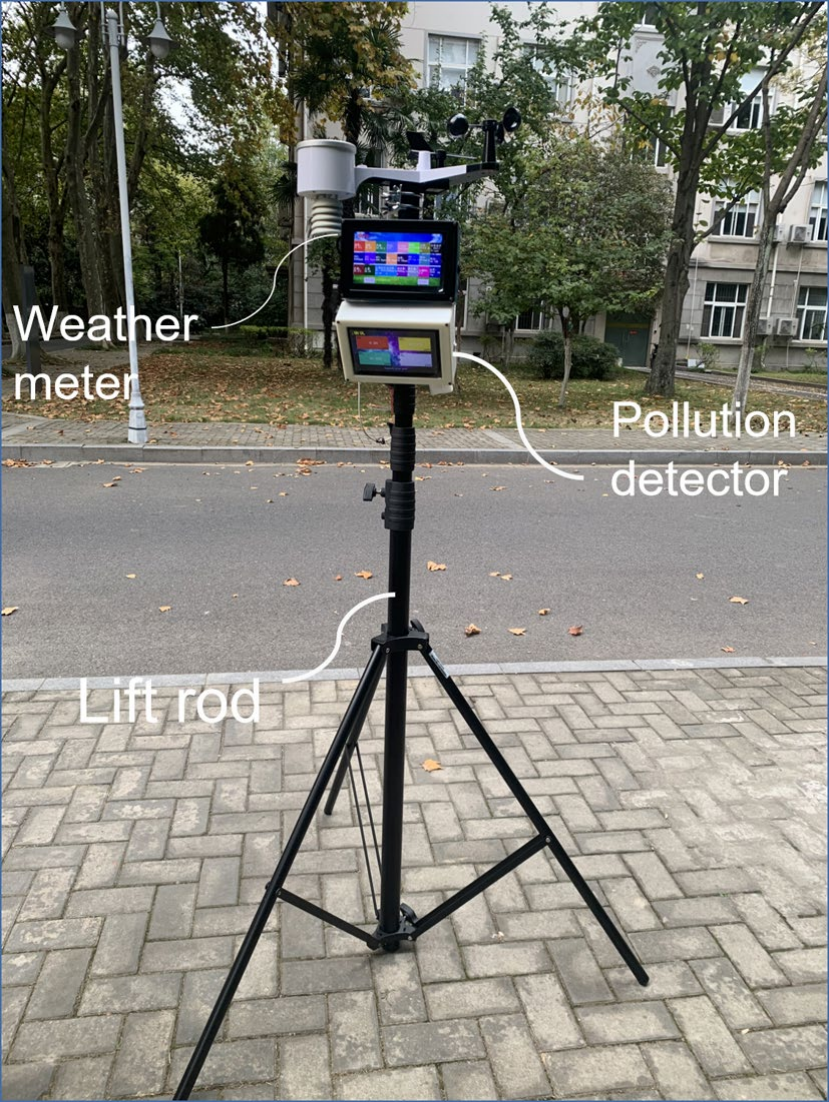
The analytical diagram of self-developed multifunctional lifting environmental detector.

The data used in this study were collected from the field. The study was conducted in the winter of 2021 and the summer of 2022, when the concentration of NO_2_ reaches its maximum and minimum in winter and summer, respectively^24,25^. The data were as follows: (1) location information, including geographic coordinates, altitude, longitude, and latitude. (2) Environmental factors including temperature, humidity, wind speed, wind direction, light, radiation, air pressure, noise and traffic flow. (3) Plant information included plant species, tree height (H), diameter at breast height (DBH), crown size, canopy area (CA) and canopy density (CD). (4) The concentration of NO_2_. Location information, environmental factors, and pollutant concentrations were recorded using a self-developed multifunctional lifting environmental detector (Table 2, Fig. 2) on weekdays without precipitation in February 2022 and July 2022. The concentration of NO_2_ was considerably lower on the rest day than on the working days^26^. The detector was mainly composed of sensors, circuit boards and related accessories. To ensure the reliability of the measurement, we went to the national automatic air quality monitoring point for ground monitoring and data comparison before the experiment every month. Taking temperature, humidity, wind speed, light, radiation, air pressure, noise, traffic flow and NO_2_ concentration as a set of data, we got a total of 1080 sets of effective data. Plant information was obtained using field surveys and measurements.

**Table 2:** The measuring range and accuracy of multifunctional lifting environmental detector.

Testing content	Measuring range	Resolution ratio	Precision	Testing content	Measuring range	Resolution ratio	Precision
NO_2_	0–100 ppm	0.01 ppm	≤ reading ± 3%	Temperature	− 40–80 °C	0.1 °C	± 2 °C
Humidity	% RH	0.1% RH	± 2% RH	Wind speed	0–60 m/s	0.1 m/s	± 0.5 m/s
Air pressure	300–1200 hpa	1 hpa	± 1.5 hpa	Humidity	0–100% RH	0.1% RH	± 2% RH
Wind speed	0–60 m/s	0.1 m/s	± 0.5 m/s	Radiation	0–2000 μw/cm^2^	1 μw/cm^2^	± 1 μw/cm^2^
Light	0–300 KLux	0.1 KLux	± 0.1 KLux	Noise	30–120 dB	0.1 dB	< 2%

**Figure 3:**
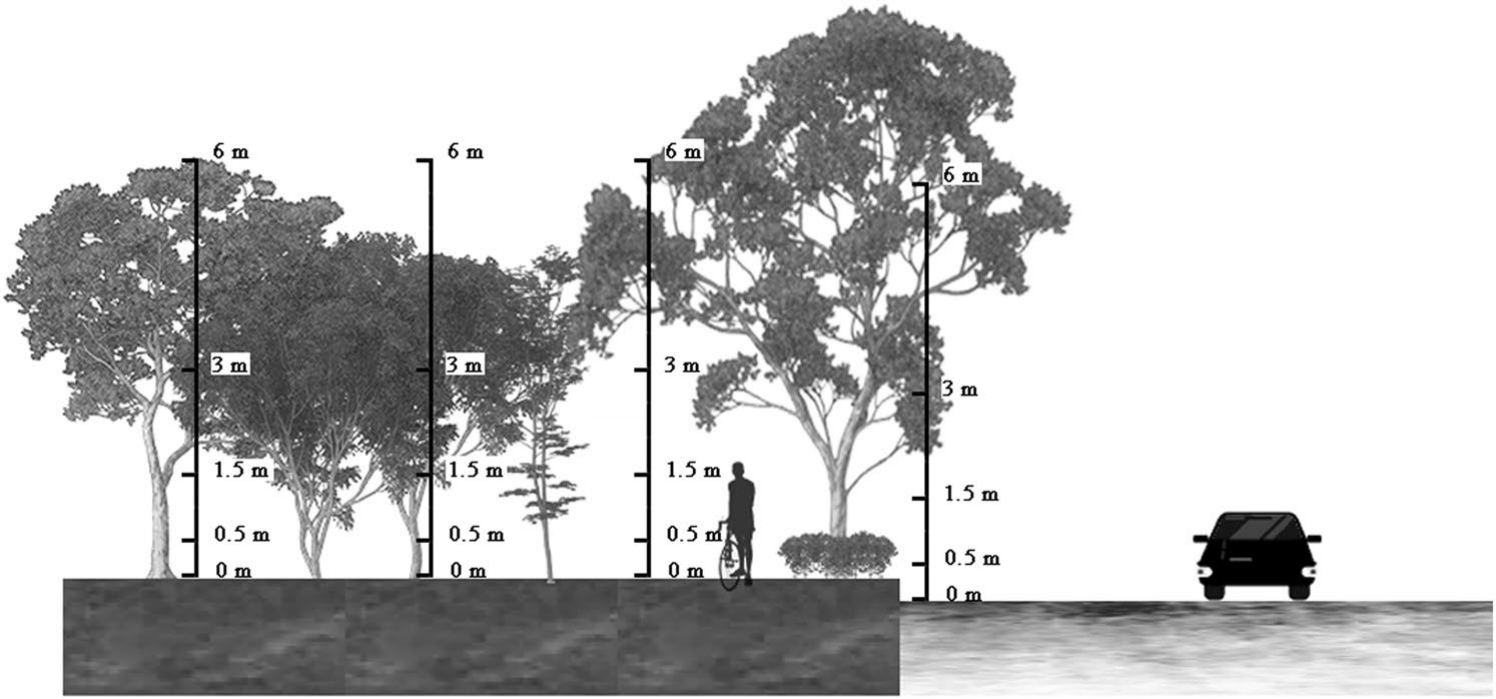
Diagram of horizontal and vertical position of multifunctional lifting environmental detector in sampling sites.

As shown in Fig. 3, a 20 × 20 m sampling site was set in the street green space, and three types of sampling points in the horizontal direction were set: the control point, sidewalk, and green space. At each sampling site, NO_2_ concentrations were recorded from 7:00 to 9:00 (morning peak), 12:00–14:00 (off-peak), and 17:00–19:00 (evening peak) on weekdays, with no precipitation and wind speeds lower than 2 m/s at heights of 0, 0.5, 1.5, 3, and 6 m above the ground.

### Data analysis

The formula for calculating the percentage of pollutant purification in the green belts of different plants is as follows:

$${\text{P}}_{{\text{n}}} = ({\text{C}}_{{\text{c}}} - {\text{C}}_{0} )/{\text{C}}_{{\text{c}}} \times {1}00\%$$

where P_n_ is the purification percentage of various pollutants by the green belt, C_c_ is the pollutant concentration on the side of the motor vehicle lane close to the green belt, and C_0_ is the green belt far from the edge of the motor vehicle. The concentration of pollutants (control concentration). * P < 0.05 and ** *P* < 0.01 were considered statistically significant and highly significant, respectively.

## Result

### Seasonal variation characteristics of meteorological factors and NO_2_ concentration in different plant communities

Comparing the meteorological factors of the plant communities in the different seasons (Table 3), it was observed that temperature, radiation, light, and noise increased, whereas air pressure decreased from winter to summer. One-way analysis of variance showed that seasonal changes significantly (*P* < 0.05) affected temperature, air pressure, and noise. It is speculated that the limitations of weather conditions on the day are more likely to affect changes in humidity, wind speed, wind direction, radiation, and light than seasonal changes.

**Table 3:** Road meteorological factor changes at the different sites.

Sites	Temperature °C)	Humidity (%)	Wind speed (m/s)	Atmospheric pressure (pa)	Wind direction	Air radiation (uw/cm^2^)	Light (Lux)	Noise (dB)
CK (winter)	6.43	42.06	0.35	102,991.45	Southeast	202.92	11,760.13	63.61
CK (summer)	30.87	74.26	0.51	99,574.3	Southwest	204.24	12,512.91	65.03
A1 (winter)	8.13	61.54	0.68	102,824.80	Southwest	564.48	22,272.59	62.92
A1 (summer)	31.79	73.27	0.89	99,572.82	Northwest	618.31	25,169.19	64.41
B1 (winter)	6.95	70.89	0.88	102,250.00	Northeast	85.35	5822.25	64.45
B1 (summer)	32.35	69.25	0.57	99,812.65	Southeast	557.60	17,939.33	65.42
C1 (winter)	7.39	76.35	0.07	102,228.91	Southeast	73.43	5858.88	61.36
C1 (summer)	30.60	77.74	0.13	100,147.52	South	116.51	6509.55	65.97
D1 (winter)	5.89	78.49	0.42	102,372.70	Northeast	46.99	4016.37	62.47
D1 (summer)	34.59	66.63	0.50	100,065.24	Southeast	480.03	20,554.07	64.10

**Figure 4:**
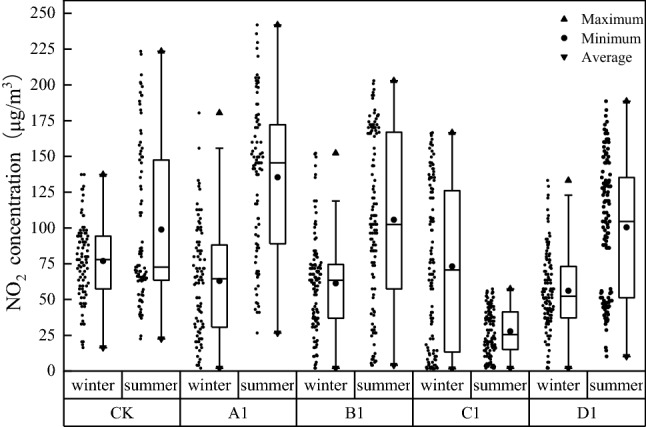
Seasonal distribution of NO_2_ concentration.

**Figure 5:**
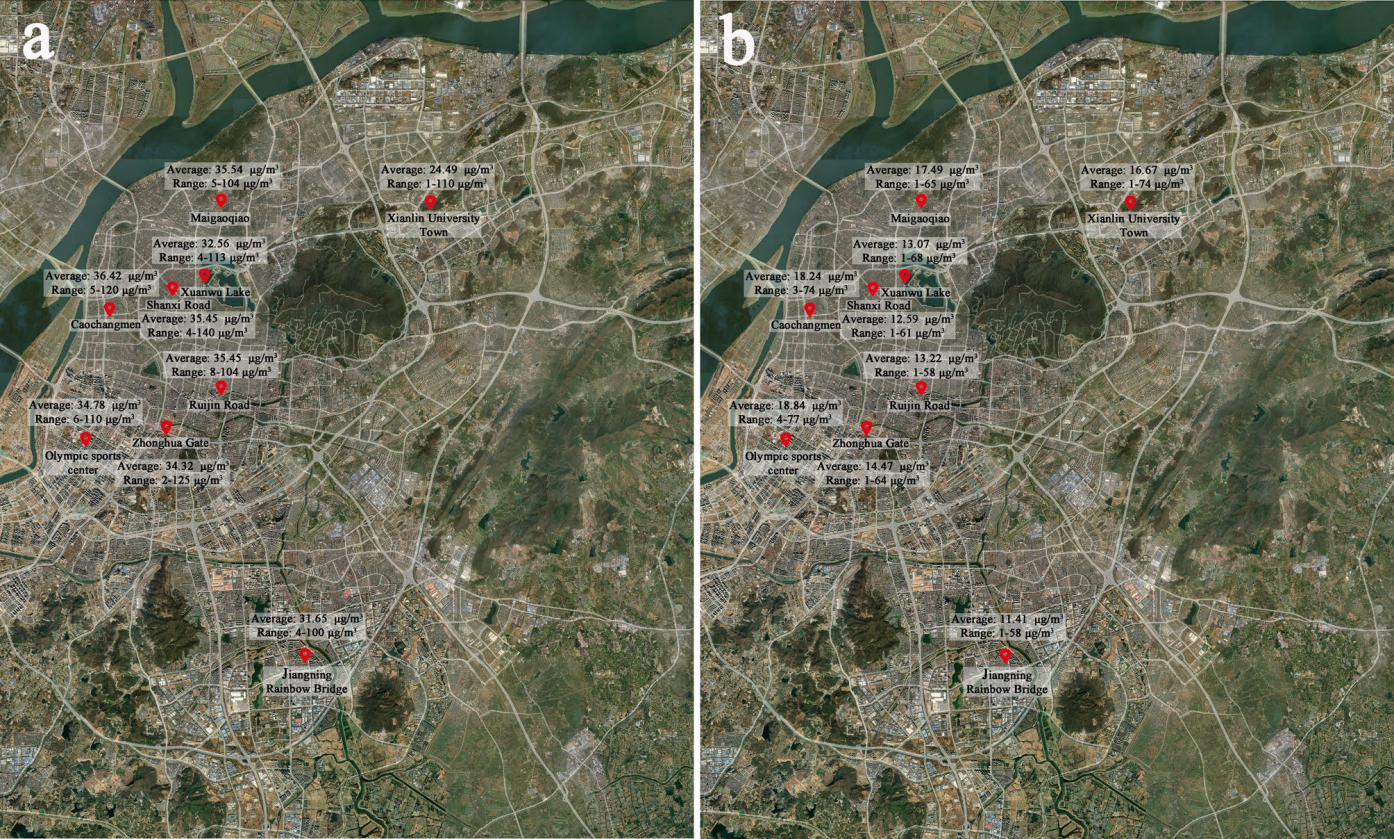
(**a**) NO_2_ of national control monitoring sites in Nanjing in February 2022 (**b**) NO_2_ of national control monitoring sites in Nanjing in July 2022 (The base images for (**a**) and (**b**) are from Google Maps. Version number: 11.60.0703. URL link: https://www.google.com.hk/maps/place/%E4%B8%AD%E5%9B%BD%E6%B1%9F%E8%8B%8F%E7%9C%81%E5%8D%97%E4%BA%AC%E5%B8%82/@32.0554234,118.67779,59275m/data=!3m1!1e3!4m5!3m4!1s0x35b58c9b668dcd83:0x8ffbb60b79df1b06!8m2!3d32.0583799!4d118.79647!5m1!1e4?hl=zh-CN).

By comparing the change in NO_2_ concentration (Fig. 4), it was found that the average NO_2_ concentration was generally higher in the summer than in the winter. The average concentration of NO_2_ in the five plant communities was higher than 10 μg/m^3^ in both summer and winter (World Health Organization average annual concentration guidelines). The average NO_2_ concentrations of CK, A1, B1 and D1 in summer were 98.93, 135.48, 105.78, 100.49 μg/m^3^, respectively. It exceeds the 24-h average level 2 concentration limit (80 μg/m^3^) set in the ambient air quality standard (GB 3095-2012). In addition, the concentrations of NO_2_ at the other sampling sites were lower than the level 2 standard concentration limit. However, by comparing the monthly data of national automatic air quality monitoring points (24-h continuous monitoring) (Fig. 5), it can be found that the average value was significantly lower than our monitoring data (6-h monitoring), and the range of values overlapped to some extent.

### Influence of environmental factors on NO_2_ concentration

**Figure 6:**
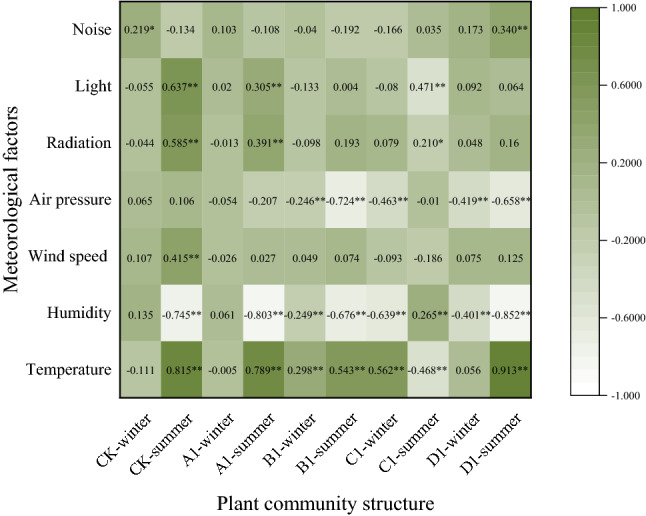
Correlation analysis between road meteorological factors and NO_2_.

The correlation analysis of meteorological factors and NO_2_ concentration found that temperature and humidity significantly (*P* < 0.01) influenced NO_2_ concentrations in winter and summer, among which temperature was significantly positively correlated while the humidity was significantly negatively correlated. Through analysis and comparison of the different seasons, it can be seen that environmental factors had a greater impact on NO_2_ concentrations in summer than in winter (Fig. 6).

As shown in Table 4, the traffic flows in winter and summer significantly (*P* < 0.05) differed between the peak and off-peak periods. However, there was no significant correlation between NO_2_ concentration and traffic flow at the five sampling sites. This indicates that the change in NO_2_ concentration on the road may be jointly affected by traffic flow and other meteorological factors.

**Table 4:** The daily traffic flow in the 5 plant communities in Nanjing.

Sites	Winter			Summer		
	Morning	Noon	Evening	Morning	Noon	Evening
CK	85/5 min	48/5 min	101/5 min	152/5 min	95/5 min	132/5 min
A1	49/5 min	28/5 min	65/5 min	42/5 min	30/5 min	45/5 min
B1	185/5 min	113/5 min	170/5 min	163/5 min	86/5 min	113/5 min
C1	169/5 min	45/5 min	146/5 min	95/5 min	26/5 min	68/5 min
D1	120/5 min	43/5 min	114/5 min	83/5 min	59/5 min	155/5 min

In light of the above, we conducted multiple linear regression analysis of temperature, humidity, air pressure and NO_2_ concentration. The results showed that in winter the fitting equation was y = 1543.36 + 0.44x_1 _− 0.81x_2 _− 0.01x_3_ R^2^ = 0.09, and in summer was y = 11330.36 + 10.25x_1 _− 1.37x_2 _− 0.11x_3_ R^2^ = 0.64, where x_1_ was temperature, x_2_ was humidity, and x_3_ was atmospheric pressure.

### Effects of different plant community structures on NO_2_ concentration

The average height through which humans breathe is 1.5 m. Considering the NO_2_ concentration at 1.5 m height, we compared and analyzed the reduction rate of NO_2_ at the five sampling sites. The reduction rate of NO_2_ in winter was D1 > C1 > A1 > CK > B1. The reduction rate of NO_2_ in the summer was D1 > C1 > B1 > CK > A1 (Table 5).

**Table 5:** Reduction rates of different plant communities.

	Plant communities	Winter (%)	Summer (%)
CK	Square	15.28	16.60
A1	Lawn	16.98	15.07
B1	Arbor-grass	10.78	22.33
C1	Arbor-shrub	21.77	23.70
D1	Arbor-shrub-grass	28.24	24.60

**Figure 7:**
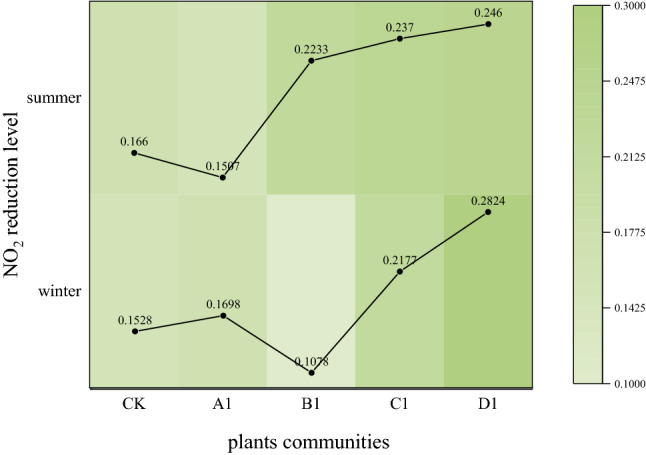
Reduction rate fluctuation of different plant communities.

As shown in Fig. 7, we analyzed the changes in the NO_2_ reduction rate at the five sampling sites and found that the reduction rate of B1 varied greatly in winter and summer (from 10.78% in winter to 22.33% in summer), indicating that the seasonal changes in plants impacted the reduction rate. The reduction rates of CK (15.28% in winter, 16.60% in summer) and A1 (16.98% in winter, 15.07% in summer) were similar and smaller than those of C1 (21.77% in winter, 23.70% in summer) and D1 (28.24% in winter, 24.60% in summer). These results indicate that road green spaces positively affect NO_2_ concentration reduction.

### Effects of plant community spatial structure on NO_2_ concentration

#### Effects of horizontal direction on NO_2_ concentration in plant community structure

**Figure 8:**
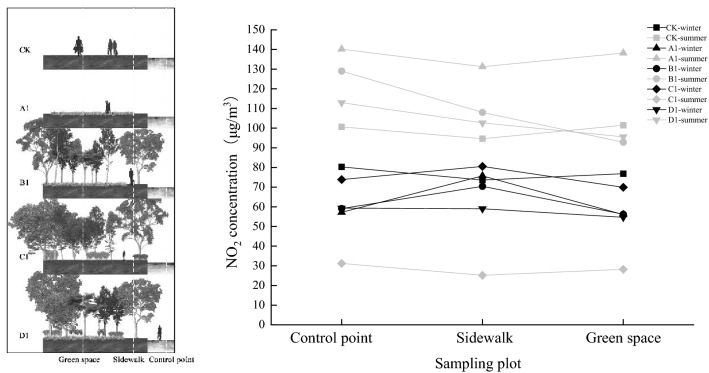
Horizontal decline rate in different plant communities.

We compared and analyzed the NO_2_ concentration in the plant communities in the horizontal direction with the control point, the sidewalk, and green space sampling points and found that the NO_2_ concentration in summer was generally higher than that in winter, and the NO_2_ concentrations in CK and A1 were higher than those in other plant community structures (as shown in Fig. 8). In CK and A1, the concentration of NO_2_ fluctuated greatly. In B1, C1, and D1, the NO_2_ concentration of the sidewalks was higher than that of the control points in winter. However, in the summer, when plants were growing vigorously, the overall performance was as follows: control point < sidewalk < green space. In general, at the sampling points with green space, the NO_2_ concentration decreased to a certain extent with an increase in road distance (especially green space < control point), indicating that road green spaces had a positive effect on the reduction of NO_2_ concentration.

#### Effect of vertical direction on NO_2_ concentration in plant community structure

**Figure 9:**
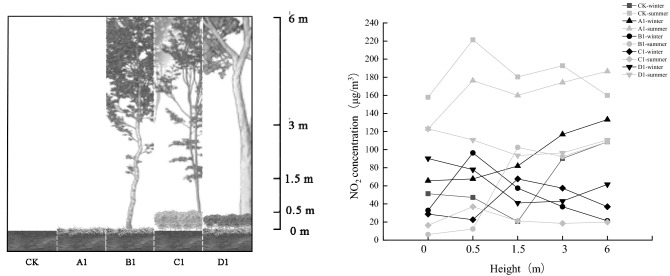
Vertical reduction rate in different plant communities.

We selected the vertical sampling points in the green space to analyze the vertical variation in NO_2_ concentration in the five plant communities and found that the vertical variation in NO_2_ concentration was inconsistent (as shown in Fig. 9). The NO_2_ concentrations in CK and A1 were higher than those in the other plant community structures. In general, NO_2_ concentration increased from 0 to 0.5 m and decreased from 0.5 to 1.5 m. C1 and D1 decreased from 0 to 0.5 m, indicating that shrubs (0–0.5 m) had a certain reduction effect on NO_2_ concentration. Overall, the NO_2_ concentration increased from 1.5 to 6 m, while B1 and C1 decreased from 1.5 to 6 m. Combined with the vertical structure diagram of the plant community shown in Fig. 7, it was suggested that the tree canopy promoted the reduction of NO_2_ concentration. However, at the branch points under the tree canopy, where there are no leaves, only the trunk will accumulate and precipitate pollutants, and thus increase the concentration.

## Discussion

### Relationship between NO_2_ concentration and environmental factors

This study found that temperature, air pressure, and noise were significantly (*P* < 0.05) correlated with seasonal changes. We also found that the average concentration of NO_2_ was generally higher in summer than in winter. This is speculated to be due to the following: 1. As the radiation and light in summer were much higher than in winter, the photochemical reactions in summer led to an increase in the secondary formation of NO_2_^27^. 2. The temperature was extremely high during the summer sampling period. Goldberg et al.^26^. showed a higher concentration of atmospheric NO_2_ at temperatures > 32 °C than at temperatures from 26 to 32 °C. In contrast, the NO_2_ concentration decreases with increasing temperature at moderate temperatures. However, July was relatively hot in Nanjing, with an average daily temperature of 30.87–34.59 °C at the sampling sites. 3. This may be related to human activity and NO_2_ emissions in the streets^28^. Study has shown that NO_2_ emissions generally peak in the summer^29^.

The correlation between the meteorological factors and NO_2_ concentration showed that temperature was mainly positively correlated with NO_2_ concentration (*P* < 0.01), whereas humidity was negatively correlated (*P* < 0.01). Other studies have shown that temperature and humidity are directly related to NO_2_ concentrations, but the correlation is not the same^3,30,31^. We believe the positive correlation between temperature and NO_2_ concentration agrees with our previous conjecture 2. When the temperature reaches a certain level, the concentration of NO_2_ increases with an increase in temperature. 

Normally, the weather factors such as wind direction and wind speed would affect pollutants attenuation to a certain extent. But it did not show significant impact in this study. This may probably because all the sampling sites were heavily affected by windbreaks and leeward, which Yin et al.^17^ and Irga et al.^20^ had also found it.

Current study suggests that vehicles are the main source of NO_2_ in cities and are markedly correlated with NO_2_ concentrations^32^. However, our study found that the changes in NO_2_ concentrations at the five sampling sites were not markedly correlated with traffic flow. Considering the above correlation, we speculated that the changes in street NO_2_ concentrations might be jointly affected by traffic flow and other meteorological factors which was also proved by our fitting results of multiple linear regression equation.

### Influence of different plant community structures on NO_2_ concentration

Desyana et al.^33^ found that vegetated areas had lower NO_2_ concentrations than that at unvegetated sites. The same finding was apparent in our study. Comparing the five sampling sites in winter and summer, we found that the reduction rate of NO_2_ in winter was as follows: D1 > C1 > A1 > CK > B1, and the reduction rate of NO_2_ in summer was D1 > C1 > B1 > CK > A1. Deciduous trees accounted for a large proportion of the vegetation at B1. We believe that after plant defoliation in winter, B1 was not open enough to facilitate air flow like A1 and CK, and could not have sufficient evergreen vegetation to purify NO_2_ like D1 and C1^34^ (as shown in Table 6). In contrast, the plant community structure with trees, shrubs, and grasses can effectively reduce NO_2_ concentrations, which is consistent with the results of Luo^35^. Rao et al.^36^ showed that NO_2_ purification by Portland trees could provide $7 million per year, which is an interesting topic. In the next phase, we plan to combine the measured pollutant data with the ecological benefits of plant communities to further explore the intrinsic value of these plant communities.

**Table 6:** Wind speed at different monitoring heights in different plant communities.

Sites/Height	0 m	0.5 m	1.5 m	3 m	6 m
	Wind speed (m/s)				
CK (winter)	0.13	0.27	0.73	0.70	0.75
CK (summer)	0.45	0.33	0.52	0.80	0.95
A1 (winter)	0.48	0.60	0.78	0.30	0.35
A1 (summer)	1.07	0.52	1.27	1.10	0.72
B1 (winter)	0.27	0.13	1.30	1.50	1.13
B1 (summer)	0.23	0.12	0.43	0.18	0.10
C1 (winter)	0.03	0.03	0.07	0.10	0.10
C1 (summer)	0.40	0.03	0.17	0.07	0.03
D1 (winter)	0.00	0.00	0.43	0.52	0.22
D1 (summer)	0.40	0.55	0.52	0.47	0.38

### Internal effects of plant community structure on NO_2_ concentration

The spatial structure of the plant community was divided into horizontal and vertical directions for comparative analysis. We found that in the horizontal direction, compared with the control point, the NO_2_ concentration was higher in the sidewalks in winter (B1, C1, and D1), which may be due to the recirculation area of airflow in front of the green space^37^. However, in summer, when the trees are growing vigorously, NO_2_ concentration gradually decreases with an increasing distance from the control point, clearly showing a trend that green space < control point. This agrees with results of Fantozzi et al.^38^, and indicates that road green spaces positively affect reducing NO_2_ concentration, especially in B1 and D1.

In the vertical direction, the concentration of NO_2_ in sampling sites with shrubs decreases from 0 to 0.5 m. Considering our current investigation and finding that the height of shrubs is less than or equal to 0.5 m, we believe that the presence of shrubs can reduce the NO_2_ concentration in green areas to a certain extent^39^. Overall, the NO_2_ concentration increased from 1.5 to 6 m, while in B1 and C1 it decreased. Considering the height of the trees under the branches in the community^40^, we believe that the arbor canopy density could promote the reduction of NO_2_ concentration, but the height of branch points under the canopy would be affected by the obstruction of air flow by the canopy, thus accumulating and precipitating NO_2_ and increasing its concentration rise (as shown in Table 6).

**Figure 10:**
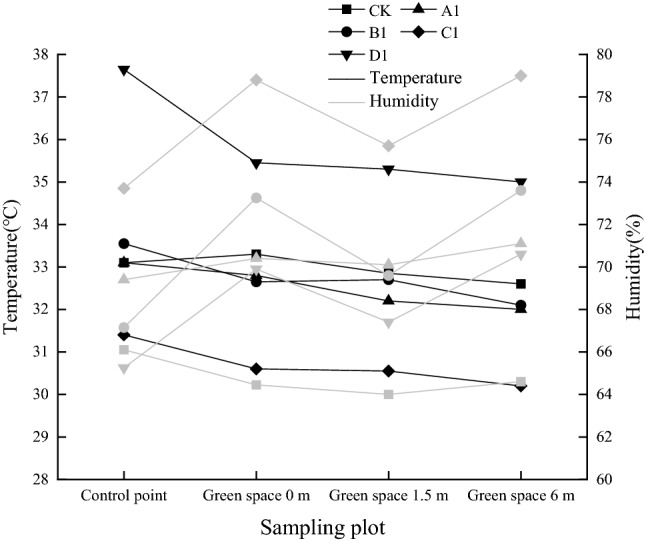
Temperature and humidity variation in different plant communities in summer.

In addition, studies have shown that green space could regulate the microclimate, especially the reduction in air temperature and the increase of air humidity in summer^41,42^. Considering the significant influence of temperature and humidity on NO_2_ concentration, we analyzed the temperature and humidity variation in different plant communities in summer (as shown in Fig. 10). The results showed that the presence of green space reduced temperature and increased humidity, which also partly explained the variation of NO_2_ concentrations in horizontal and vertical direction.

## Conclusion

In this study, we investigated the effects of five small plant communities along urban streets on NO_2_ concentrations in the atmosphere. The results showed that temperature, air pressure, and noise were significantly correlated with seasonal changes, and the average NO_2_ concentration in summer was generally higher than that in winter. The correlation between environmental factors and NO_2_ concentration showed that temperature and humidity were significantly (*P* < 0.01) correlated with NO_2_ concentration; however, the change in NO_2_ concentration in the streets may be jointly affected by traffic flow and other meteorological factors. By comparing different plant community structures and the internal space of NO2 concentration changes, we found that the plant community structure with arbors, shrubs, and grass was more effective in reducing pollution, and evergreen plants were more effective. These findings will help predict the impact of plant communities on NO_2_ concentrations in urban streets and help city managers and planners effectively reduce NO_2_ pollution in the air.

Since our study focused on winter and summer, we plan to continue the monitoring of pollutants until the length of one year in the future to observe the effects of environmental factors and vegetation changes on pollutant concentrations. In addition, we will import the data of five sites into the i-Tree Eco model to evaluate their ecological benefits and analysis the difference between model calculation of pollutant purification and actual monitoring.

## Data Availability

The datasets generated and analysed during the current study are not publicly available due [The conclusion of National Natural Science Foundation of China] but are available from the corresponding author on reasonable request.
